# Protein kinase R-like ER kinase and its role in endoplasmic reticulum stress-decided cell fate

**DOI:** 10.1038/cddis.2015.183

**Published:** 2015-07-30

**Authors:** Z Liu, Y Lv, N Zhao, G Guan, J Wang

**Affiliations:** 1 Department of Cardiology, Shaanxi Provincial People's Hospital, Xi'an, China

## Abstract

Over the past few decades, understandings and evidences concerning the role of endoplasmic reticulum (ER) stress in deciding the cell fate have been constantly growing. Generally, during ER stress, the signal transductions are mainly conducted by three ER stress transducers: protein kinase R-like endoplasmic reticulum kinase (PERK), inositol-requiring kinase 1 (IRE1) and activating transcription factor 6 (ATF6). Consequently, the harmful stimuli from the ER stress transducers induce apoptosis and autophagy, which share several crosstalks and eventually decide the cell fate. The dominance of apoptosis or autophagy induced by ER stress depends on the type and degree of the stimuli. When ER stress is too severe and prolonged, apoptosis is induced to eliminate the damaged cells; however, when stimuli are mild, cell survival is promoted to maintain normal physiological functions by inducing autophagy. Although all the three pathways participate in ER stress-induced apoptosis and autophagy, PERK shows several unique characteristics by interacting with some specific downstream effectors. Notably, there are some preliminary findings on PERK-dependent mechanisms switching autophagy and apoptosis. In this review, we particularly focused on the novel, intriguing and complicated role of PERK in ER stress-decided cell fate, and also discussed more roles of PERK in restoring cellular homeostasis. However, more in-depth knowledge of PERK in the future would facilitate our understanding about many human diseases and benefit in searching for new molecular therapeutic targets.

## Facts

- Protein kinase R-like endoplasmic reticulum kinase (PERK) is one of the major transducers of endoplasmic reticulum (ER) stress, participating in regulating fundamental cell functions.
- Composed of the cytosolic and kinase domains, ERK is activated in a lined-up formation after interaction with the peptide chain of an unfolded/misfolded protein.
- Signals of apoptosis and autophagy induced by ER stress could be transducted through PERK pathway to trigger cell death or to maintain cell survival.
- There is a switching mechanism between autophagy and apoptosis, which is regulated by PERK.

## Open Questions

- How to explain the finding that PERK deficiency presented stronger protective effect against apoptosis rather than activating transcription factor 6 (ATF6) or inositol-requiring kinase 1 (IRE1) deletion?
- Are there any other downstream pathways of PERK when inducing cell apoptosis and autophagy?
- What are the exact interaction mechanisms and significance of crosstalk network among PERK-governed pathways in ER stress?

ER was spotted by KR Porter, A Claude and EF Fullam in 1945 in cultured mice fibroblasts.^1^ ER was found in almost all kinds of mammalian eukaryotic cells (except mature red blood cells). ER has been considered as a tubular system composed of a biomembrane, providing pathways for intracellular material transportation and a platform for various biological enzymatic reactions.^2^ ER is considered as one of the major vital organelles because it executes and participates in various fundamental biological functions including protein synthesis, steroid hormone synthesis, posttranslational protein modification, peptide chain folding, glucose concentration regulation, calcium homeostasis and lipid metabolism.^3, 4, 5^

ER is critically involved in protein processing, which was described as a 'protein folding factory'. By coordinating activities of protein folding/modifying enzymes and interactions of molecular chaperones, ER optimizes the microenvironment for posttranslational processing of nascent proteins.^6^ Correctly folded and modified proteins were then transported to the Golgi complex, which is responsible for protein maturation and distribution. The unfolded and misfolded proteins were deleted by ER-associated degradation (ERAD) system and cytosolic proteasomes.^7^ However, certain pathological conditions, such as extracellular blood glucose concentration fluctuation, oxidative stress, toxic agents/drug and radioactive radiation, would lead to overproduction of unfolded/misfolded proteins. The accumulation of these unfolded/misfolded proteins in ER lumen aggravates the burden of ER quality control system and eventually initiates unfolded protein response (UPR).^8^

The UPR was first described by Kozutsumi *et al.*^9^ in the 1980 s in mammalian cells in response to stressful conditions.^9^ UPR was considered a protective response to suspend protein translation massively but to induce some specific gene translations, which intend to restore the ER protein folding capacity. The transcription and translation of several ER-resident proteins, such as glucose-regulated protein 78 (GRP78), which belongs to heat-shock protein 70 (Hsp70) family, was markedly upregulated.^10^ Similar phenomenon was also observed in yeast where the expression of Kar2p, a GRP78 homolog, was increased too.^11^ However, when the external stressful factors fail to withdraw, the accumulation rate of misfolded proteins in the ER lumen would overwhelm the handling capacity of the ERAD system, initiating a pathological cellular stressful condition called ER stress.

## Downstream Signaling Pathways of UPR Sensors

### Main signaling arms of ER stress

Generally, the harmful signaling was considered mainly sensed by three ER-resident transmembrane molecules, which were referred to as ATF6, IRE1 and PERK.^12^ As one of the most highly expressed ER chaperones, GRP78 was known to bind to the hydrophobic domains of proteins with its C-terminal substrate-binding domain to protect protein against misfolding. This process was thought energy consuming because GRP78 is actually an ATPase for its N-terminal ATPase domain.^13^ In resting cells under physiological conditions, the activities in ATF6-, IRE1- and PERK-governed signal-transduction branches were blocked when GRP78 binds to their lumenal domains.^14^ However, when affected by sustained harmful stress, GRP78 would disassociate from these sensors to initiate ER stress. ATF6 is activated after being cleaved, whereas PERK and IRE1 are activated by self-transphosphorylation. It was reported that the sensitivities to external stressors vary among these signaling branches. The activation of PERK-governed signaling was almost simultaneous with the onset of ER stress (GRP78 disassociation), followed by activation of ATF6 and then IRE1 signaling pathways.^12, 15^

### PERK is one of the major signaling pathways of ER stress

#### ATF6

ATF6 is a type-II transmembrane protein located on the ER. The stress signal could be sensed by lumenal C-terminal region of ATF6 right after exposure to stressful conditions rapidly. After the disassociation from GRP78, ATF6 translocates to the Golgi complex where the full-length ATF6 is cleaved by site-1 and site-2 proteases.^16^ The generated 50 kDa fragment is bioactive because it contains the bZIP nuclear transcription activation domain that originated from the N-terminal region of ATF6. This fragment, also referred to as ATF6 P50, then translocates to the nucleus to regulate survival-related gene expression such as C/BEP homologous protein (CHOP) and X-box-binding protein 1 (XBP1).^17^

#### IRE1

The endoribonuclease domain and the Ser/Thr kinase domain endow IRE1 dual enzymatic activities. The activation of IRE1 signaling was found delayed as the transcription of its substrate XBP1 mRNA is upregulated after activation of the ATF6 signaling pathway.^18^ After activation by self-phosphorylation, right after dissociation from GRP78, the phosphorylated IRE1 introduces an unconventional splicing by removing an intron to generate a spliced variant of XBP1, which is called sXBP1.^19^ It has been proved that sXBP1 had a part in regulating transcriptions of ER stress proteins, which were found involved in assisting protein folding, maturation and transportation, as well as degrading misfolded proteins.^20^ Furthermore, IRE1 was also considered a key initiator of ER stress-induced cell death.^21^ According to several recent studies, cell death was promoted by regulated IRE1-dependent decay of mRNA, which is a process targeting and degrading protein folding-related mRNAs.^22^

#### PERK

The *α*-subunit of eukaryotic initiation factor 2 (eIF2*α*) phosphorylation is executed by eIF2*α* kinase family, which is considered protective in cells engaging various stimuli by biasing general protein synthesis but initiating stress-related protein production. As most transcription processes are shut down, ER lumen protein accumulation is, therefore, attenuated and the ER stress is relived. It is widely accepted that there are mainly four members of eIF2*α* kinase family, namely PERK, protein kinase double-stranded RNA-dependent, heme-regulated inhibitor and general control non-derepressible-2. A wide range of cellular stress, such as heme deprivation, UV irradiation, amino-acid starvation and viral infection, would activate these kinases, which subsequently trigger the phosphorylation of eIF2*α*.^23^ In ER stress, right after disassociation of GRP78, the activation of PERK signaling pathway is initiated upon its dimerization and autophosphorylation.^24^ Then, eIF2*α*, which is one of the PERK's downstream effectors, is phosphorylated to suppress the general gene translations by inhibiting ribosome transportation of initiator methionyl-tRNAi^Met^.^25^ Notably, translations of genes containing internal ribosomal entry sites were selectively enhanced after phosphorylation of eIF2*α*.^26^ The canonical example is the *ATF4* (activating transcription factor 4) gene encoded mRNA. The translation of ATF4 after scanning ribosome association is a process depending on eIF2-GTP/Met-tRNA_i_^Met^ levels, which is reduced during ER stress.^27, 28^

## The Molecular Structure and Activation of PERK

**Figure 1:**
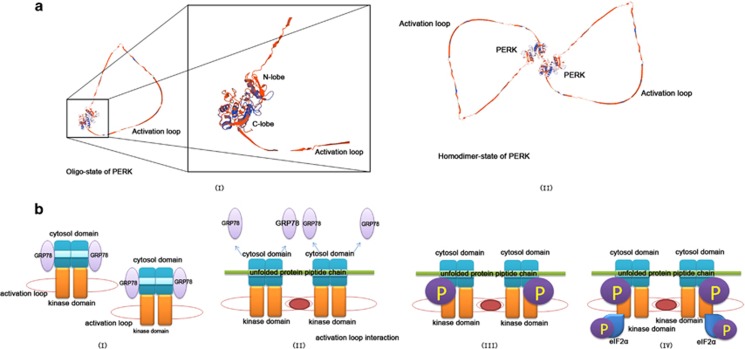
Molecular structure of PERK kinase domain and 'line-up' activation mechanism. (**a**) The presumed three-dimensional (3D) molecular model of PERK kinase domain. (I) The oligostate of PERK kinase domain. This domain could be divided into N-lobe and C-lobe, which are connected by a short hinge loop. There is a distinctive long activation loop in this structure. (II) Two individual PERK molecules dimerize to establish the homodimer state of PERK kinase domain, which is the most common form of PERK. (**b**) (I) At resting state without ER stress, GRP78 binds to the cytosol domain of dimerized PERK. (II) Under external harmful stimuli, the unfolded protein accumulate in the ER lumen to initiate ER stress by disassociating GRP78 from PERK. Then, the peptide chain of an unfolded protein bind to MHC-like grooves of the cytoplasmic domain to 'line up' the stacked PERK dimers. (III) The activation loop of neighboring PERK kinase domain interact with each other to trigger autophosphorylation of PERK. (IV) eIF2*α* are recruited and phosphorylated by activated PERK

PERK is a protein kinase that belongs to eIF2*α* kinase subfamily. PERK is composed of the cytoplasmic and kinase domains. The cytoplasmic domain senses the accumulation of unfolded/misfolded proteins in the ER lumen. After stimulation, PERK is activated by autophosphorylation of its kinase domain and acquired full catalytic activity to further phosphorylate eIF*α* at Ser51 specifically.^29^ Similar to most typical protein kinases, the structure of the kinase domain contains a C-terminal lobe (C-lobe) and an N-terminal lobe (N-lobe). There is a short hinge loop linking the two lobes. The N-lobe comprises three *α*-helices and five *β*-strands, whereas the C-lobe comprises two short *β*-strands, seven *α*-helices and a long activation loop^30^ (Figure 1a).

Located on the ER membrane, PERK is presented as a homodimer. The cytoplasmic domain is binded by the ER chaperone GRP78 under ER stress-free conditions. ER stress initiation would facilitate the disassociation of GRP78 from the cytoplasmic domain. Then, the unfolded/misfolded proteins in the ER lumen bind to MHC-like grooves of the cytoplasmic domain and subsequently trigger stack of PERK homodimers. Along the polypeptide of unfolded/misfolded proteins, the PERK dimers are lined up.^30^ This steric configuration allows the activation loop of one dimer reach the catalytic site of the other dimer to cause self-phosphorylation. Then, the downstream molecules of PERK are recruited and phosphorylated (Figure 1b).

## Role of PERK in ER Stress-Mediated Cell Apoptosis

**Figure 2:**
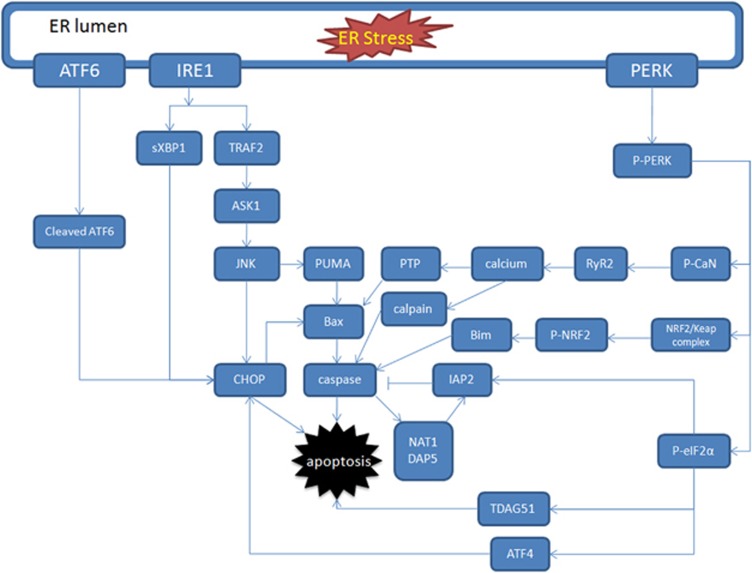
Schematic diagram of signal-transduction network during ER stress-induced cell apoptosis. Apoptotic signals are transduced through ATF6-, IRE1- and PERK-governed signaling pathways in ER stress-induced apoptosis. This diagram mainly showed PERK-associated pathways, whereas ATF6- and IRE1-regulated pathways are briefly demonstrated. According to previous studies, in ER stress-induced apoptosis, PERK/eIF2*α*/ATF4, PERK/CaN, PERK/eIF2*α*/TDAG51, PERK/eIF2*α*/IAP2 and PERK/NRF2 pathways are considered PERK-associated

In response to excessive ER stress, proapoptotic events are intended to be induced. Although there are still debates and arguments around molecular events in stress-induced apoptosis, a number of cell death-related signaling pathways are involved in Figure 2.

### Shared pathways

After exposure to sustained and excessive ER stress, the above-mentioned three branches, IRE1, PERK and ATF6 signaling, would lead to programmed cell death through several common pathways. CHOP and caspase-mediated apoptotic pathways are the most studied ones. These two pathways are not exclusive and even closely connected. In most cases, CHOP-induced apoptosis is the form of caspase-dependent cell death. However, there are still other unique molecules that receive the apoptotic signaling transduced by CHOP.

#### CHOP

As a major component of ER stress-induced apoptosis, CHOP-mediated pathway is the most described and characterized pathway in ER stress-induced cell death. CHOP is also known as GADD153 (growth arrest and DNA damage 153), whose activation is induced by ATF6 and PERK.^17, 31^ It is now generally accepted that CHOP is a major molecule regulating ER stress-induced apoptosis. Apoptotic molecules such as death receptor 5, GADD34 and ER oxidase 1 and so on are considered the target of CHOP.^32, 33^ As CHOP-related apoptotic signaling network is complicated, more investigations are still needed to interpret the exact mechanism.

#### Caspase cascade

Caspase cascade activation is a well-known proapoptotic event that is also involved in ER stress-induced apoptosis.^34^ Several members, including caspase-2, caspase-4 (human), caspase-12 (rodents), caspase-3, caspase-7 and caspase-9, were believed to have roles in this process. It was suggested that caspase-12 (caspase-4) was specific to death signals in ER stress rather than other cell death mechanisms.^35, 36^ Caspase-12 (caspase-4) is dispensable for cell death but crucial for ER-stress-induced apoptosis. Caspase-2 (caspase-4) was found located on the ER membrane, the activation of which would lead to caspase-9/caspase-3 activation, resulting in programmed cell death.

### PERK-associated pathways

The three branches of ER stress, PERK, IRE1 and ATF6, show their uniqueness when transducing survival signals in ER stress. As a central regulator of ER stress, PERK decides cell fate by interacting with its own downstream molecules to form corresponding pathways (Table 1).

#### eIF2*α*/ATF4

Upon activation of PERK in ER stress, 80S ribosome assembly and massive protein synthesis are inhibited. This inhibition effect was believed to be due to phosphorylation of eIF2*α* by activated PERK,^26, 37^ which would conduct proapoptotic signaling. On the other hand, the dephosphorylation of eIF2*α* could suppress PERK signaling by conducting prosurvival signaling. For example, a recent research showed that after eIF2*α* was dephosphorylated by GADD34, the downstream ATF4/CHOP signaling was suppressed.^38^ Therefore, eIF2*α* could be treated as one of the critical junctures in PERK-mediated signaling.

Unlike most genes, *ATF4* is characterized by its upstream open reading frames (ORFs) located on the 5'-untranslated region. Thus, these ORFs enable ATF4 escape from massive gene translation suspension, whereas the translation of ATF4 is often ceased under normal conditions without ER stress.^39, 40^ As a member of cAMP-responsive element-binding protein family, ATF4 has a broad spectrum of targeted molecules. It was documented that ATF4 acted as a prodeath factor under ER stress.^41^ The cell death-inducing function of ATF4 is attributed to the initiation transcriptions of apoptotic factors including CHOP and Noxa.^42^ Noxa is one of BH3-only proteins, which are found as inhibitors of prosurvival proteins such as Bcl-2 and activators of proapoptotic proteins such as Bak and Bax.^43^

#### Cellular inhibitor of apoptosis

IAP is a gene family that encodes IAP proteins, which were initially found in host cells undergoing baculoviral infection. IAPs were described critical in mediating host cell viability.^44^ The most frequently described membranes of cellular inhibitor of apoptosis (cIAP) proteins are cIAP1, cIAP2 and X-chromosome-linked IAP (XIAP).^45^ Previously, it has been shown that in response to stimuli from ER stress, expression of cIAP1, cIAP2 and XIAP was elevated, which was protective against cell death.^46^ cIAP machinery in ER stress was reported in a PERK-dependent manner.^46^ The increased cIAP expression was found to delay the onset of ER stress-induced apoptosis. Caspase cascade is activated upon ER stress and further cleaves molecules such as *N*-acetyltransferase 1 (NAT1) and death-associated protein 5 (DAP5).^47, 48^ The resulting cleavage products would in turn activate internal ribosome entry site of IAP2, elevating the translation of IAP2. It was demonstrated that IAP silencing increased the sensitivity to ER stress-induced apoptosis.

#### T-cell death-associated gene 51

T-cell death-associated gene 51 (TDAG51) is a member of pleckstrin homology-related domain family, which was found as a proapoptotic factor promoting cell apoptosis.^49^ The central motif of TDAG51 consists of pleckstrin homology domain and several C-terminal proline-glutamine/histidine repeats. This characterized structure endows TDAG51 the activity of transcriptional regulation, participating in inducing apoptosis. The activation of PERK/eIF2*α* would lead to the inhibition of most mRNA translation but inducing several gene expressions, including GRP78, CHOP and TDAG51.^50^ Although not fully clarified, previous studies showed that the activation of TDAG51 is an ER stress-induced eIF2*α*-dependent process.^51^ Thus, it is reasonable to speculate that TDAG51 is one of the downstream effector of PERK regulating cell fate.

#### Nuclear respiratory factor

Nuclear respiratory factors (NRFs) are members of the CNC-basic leucine zipper (CNC-bZIP) family of transcriptional factors. The structures of NRFs are highly conserved. In resting cells, by associating with cytoskeletal anchor protein Kelch-like Ech-associated protein 1 (Keap1), NRF2 is resident in latent cytoplasmic complexes.^52^ Results from previous work demonstrated that NRF2 was one of the direct substrate molecules of PERK.^53, 54^ PERK-dependent phosphorylation is sufficient and necessary to initiate the separation of NRF2 from Keap1 with the assistance of NRF1.^55^ NRF2−/− cells were found more sensitive to ER stress- induced apoptosis,^56, 57^ indicating that NRF2 is a antisurvival factor.

#### Tribbles homolog 3

Tribbles homolog 3 (TRIB3) refers to the mammalian homolog of *Drosophila* tribbles and draw attention because of its correlation with cell death. TRIB3 was initially located in neuronal cells undergoing apoptosis as a cell death-inducible protein kinase.^58^ In ER-stressed cells, TRIB3 expression was elevated. Further investigation showed that TRIB3 interacted directly with CHOP without influencing the expression level of CHOP, whereas CHOP overexpression activated the promoter activity of TRIB3.^59^ The promoter region pTRIB3-Luc1, 2, 5 and 6 was identified as the ER stress response element with a CHOP binding site inside. According to previous studies, the expression of TRIB3 was induced by the PERK/ATF4 pathway.^60^ Notably, TRIB3-silenced cells showed stronger resistance to ER stress-induced apoptosis. These results indicate that TRIB3 is a PERK/ATF4-inducible proapoptotic factor in ER stress.

#### CaN/FKBP12.6

It was suggested that cytosolic calcium elevation would lead to the opening of mitochondrial permeability transition pore, which resulted in subsequent loss and collapse of mitochondrial membrane potential and thus triggered the intrinsic apoptosis pathway.^61^ ER is recognized as the cellular 'calcium pool', and under physiological conditions, opening of ER-associated calcium channels are inhibited. For example, FK506-binding protein 12.6 (FKBP12.6) binds to ryanodine receptor2 (RyR2) calcium channel to inhibit calcium outflow from ER under normal physiological conditions.^62^ However, under certain pathological conditions, depletion of ER calcium store was found in cells undergoing ER stress.^63^ In our previous study, we demonstrated that after induction of ER stress, autophosphorylated PERK increased the enzymatic activity of calcineurin (CaN) by direct interaction. CaN then facilitated the disassociation of FKBP12.6 from RyR2 calcium channel to trigger calcium outflow from the ER to the cytosol.^64^ This Can/FKBP12.6 pathway-regulated calcium overloading could be considered as one of the possible mechanisms of ER stress-induced apoptosis.

## Role of PERK in ER Stress-Induced Autophagy

It is now generally accepted that almost all cells are undergoing process of autophagy at the basal level. Normal cellular function is maintained by the basal autophagy, which self-digests damaged organelles and proteins by lysomome to provide nascent amino acids and nucleic acids for organelle repair and protein regeneration. Pathological conditions such as oxidative stress and viral infection would initiate autophagy.^65^ The isolation membrane is formed at the preautophagosomal site, which is composed of Atg proteins. Then, the isolation membrane (autophagic membrane) is elongated to form autophagosome with the assistance of ubiqutin-like protein conjugation systems, namely the LC3 and Atg12 protein systems, which modify autophgic proteins such as Atg8 and Atg5. An autolysosome where self-digestion occurs is eventually formed by fusion of autophagosome and lysosomal/endosomal vesicles. It was believed that endoplasmic reticulum served as a platform for the initiation of autophagy at the subcellular level.^66^ Furthermore, more and more correlations between three arms of ER stress and autophagy were found in recent studies.^67, 68^ Lines of evidences suggested that there were autophagy-dependent cell death under ER stress conditions.^69, 70, 71^ Therefore, signaling in ER stress could be the potential switching mechanism between autophagy and apoptosis.

### Autophagy is regulated by ER stress pathways

Upon activation of signal transductions in ER stress, the autophagic gene were found as targets of ER stress-related transcriptional factors^72, 73, 74, 75, 76, 77, 78^ (Table 2). Mutant huntingtin aggregation-induced cytotoxicity in neurons was one of the critical mechanisms of Huntington's disease. These accumulated mutant huntingtin could be eliminated by autophagic flux but which was found impaired by IRE1/TRAF2 signaling under circumstances of ER stress.^79^ By phosphorylating and dissociating Beclin1 from Bcl2, death-associated protein kinase 1 (DAPK1) promotes autophagy.^80^ ATF6 was found as an autophagy-positive regulator by interacting with ERK1/2 target site of C/EBP-β and form a heterodimer that activates the promoter of DAPK1.^81^ PERK-governed pathway was also found to have a role in ER stress-induced autophagy.

### The involvement of PERK in ER stress-induced autophagy

#### eIF2*α*/ATF4 pathway

According to results from several previous studies, during ER stress, eIF2*α*/ATF4 signaling pathway had an important role in inducing and regulating autophagy.^75^ Activation of PERK pathway is crucial for autophagic flux, which maintains cell survival. In yeast and mammals, phosphorylation of eIF2*α* is required in inducing autophagy. During the autophagic induction phase, several eIF2*α* phosphorylation-dependent selective translation would lead to Atg12 upregulation, which then stimulates the formation of Atg5–Atg12–Atg16 complex, resulting in the promotion of LC3-I to LC3-II conversion.^82^ Moreover, it was reported that ATF4 activity was essential for some autophagic gene transcription.^83^ In a recent study, by chromatin immunoprecipitation analysis (ChIP) analysis, sets of autophagic genes including *Atg3*, *Atg12*, *Atg16*, *Map*, *Becn1* and *Gabarapl2* were identified as targets for ATF4.^75^ Meanwhile, transcriptions of autophagic genes including *Sqstm1, Nbr1 and Atg7* were boosted when ATF4 and CHOP were coactivated.^75^

#### AMPK/mTORC1 pathway

Regulation of autophagy through modulation of the AMP-activated protein kinase (AMPK)/mammalian target of rapamycin C1 (mTORC1) pathway by PERK was found in endothelial cells during ECM detachment.^84^ mTOR functions as a protein kinase coordinating multiple cellular metabolic processes.^85^ It is believed that mTOR is a typical negative autophagic regulator.^86^ mTORC1 is a complex that comprises mTOR, Raptor and mLST8. Inhibition of mTOR is considered to promote autophagy by regulating Atg1 in yeast and Atg1 complex, ULK (UNC-51-like kinase), in mammals. External stress such as ATP:ADP ratio reversion would lead to the activation of AMPK, which then inhibits the mTOR activity to initiate autophagy.^87^ In endothelial cells, under ER stress induced by ECM detachment, AMPK was found to be activated and induces autophagy by rapidly reproducing ATP to promote cell survival. Importantly, PERK was identified as the upstream activator of AMPK phosphorylation at the AMPK site, which is a substrate for LKB1.^84^ However, more detailed mechanisms concerning this process are still under investigation. The PERK/AMPK/mTORC1 pathway-mediated autophagy was found only in endothelial cells. More studies are needed to clarify whether this is a universal mechanism.

### PERK pathway switches signal transduction from autophagy to apoptosis

It may seem contradictory that PERK/eIF2*α*/ATF4 pathway participated in both ER stress-induced apoptosis and autophagy, but recent studies in selenite-treated leukemia NB4 cells indicated that it was the PERK/eIF2*α*/ATF4 axis that itself had modulated the switch between autophagy and apoptosis with the assistance of p38.^88^ It was reported in the same study that PERK modulated the phosphorylation of p38, which affected the target selection of ATF4 to mediate the switch between apoptosis and autophagy. P38 was considered the critical factor linking the crosstalk between autophagy and apoptosis by modulating PERK/eIF2*α*/ATF4 signaling.^88^

Under physiological conditions, ER stress-induced intracellular autophagy is maintained at the basal level. In selenite-induced autophagy in Jurkat cells, by interacting with promoters of autophagic genes, PERK/ eIF2*α*/ATF4 pathway induces autophagy. During this progression, the binding of ATF4 to autophagic gene such as Map1LC3B is directed and promoted by phosphorylated eIF4E.^89^ However, under pathological conditions, after ER stress is aroused, this pathway would modulate to shut down autophagy and to initiate apoptosis.

**Figure 3:**
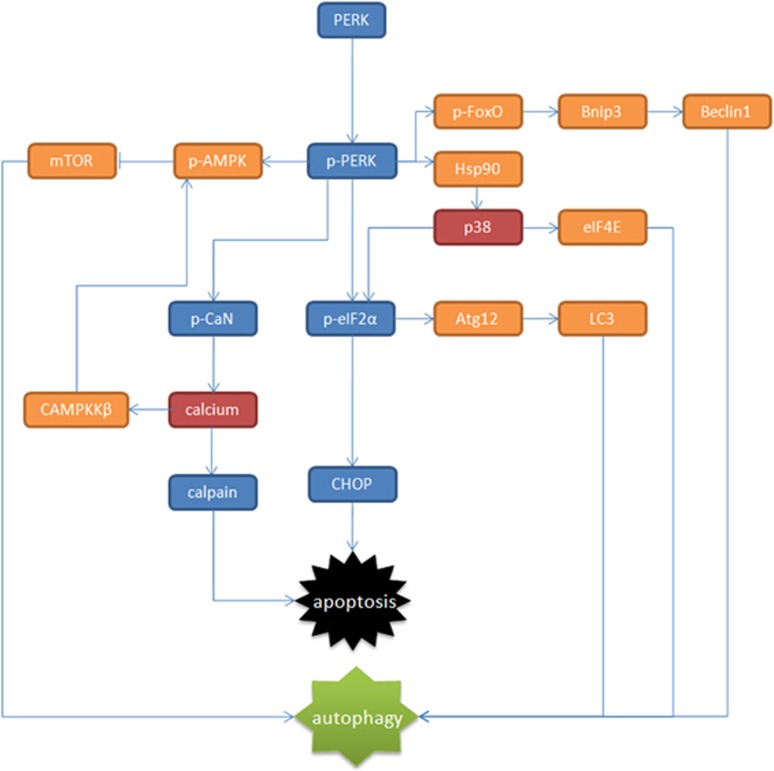
Brief diagram of PERK-dependent pathways in ER stress-induced autophagy and switching mechanism between apoptosis and autophagy. In this figure, pathways marked in blue indicated the pathways resulting in apoptosis; pathways marked in orange indicated pathways leading to autophagy. Molecules marked red (namely p38 and calcium) indicate the key regulator of PERK's switching role between autophagy and apoptosis during ER stress.

The dual role of p38 as an apoptotic promoter and an autophagic initiator is possible because of the abundance of p38 upstream regulators. It was reported that in human pancreatic cancer cells, PERK activation decreases the expression of Hsp90 and Hsp90–Cdc37 chaperone complex suppressed the autophosphorylation of p38.^90^ Under circumstances of ER stress, in the autophagy-to-apoptosis switching process, activated PERK inhibits the activity of Hsp90–Cdc37 complex to initiate the p38 autophosphorylation. Activated p38 would suppress the phosphorylation of eIF4E but increase the phosphorylation of eIF2*α*.^88^ The docking sequence for ATF4, CRE-like elements, locates in promoters of both CHOP and MAP1LC3B. Thus, ATF4 would be more inclined to initiate the binding with CHOP promoter to induce transcription of proapoptotic genes to trigger programmed cell death.^88^ The switching mechanism is demonstrated briefly in Figure 3.

## Perspectives

As one of the important intracellular organelles, ER participates in executing and regulating multiple cellular fundamental biological functions. Under certain physiological or pathological conditions, as the unfolded/misfolded proteins accumulating in the ER lumen, ER stress is induced. Currently, ER stress is considered as a cell fate-deciding event. If cells are unable to compensate under severe external stimuli, cell apoptosis would be induced by ER stress. If the stimuli are mild, ER stress-induced autophagy would be introduced to promote cell survival by providing nutrients via self-digesting damaged organelles and proteins. This sophisticated but complicated mechanism ascertains the stability of the inner biological environment and strengthens the protective responses to harmful external stimuli.

PERK is an ER-resident protein that mediates signal transduction during ER stress. Along with ATF6 and IRE1, PERK is also recognized as one of the main transducers of ER stress. The death/survival signals transduced in ER stress are sensed by these transducers to trigger either apoptosis or autophagy. However, according to several previous studies of our team and others, there are some uniqueness of PERK compared with ATF6- or IRE1-governed signaling pathways in inducing apoptosis and autophagy.

In high-glucose-mediated ER stress-induced myocytes apoptosis, we found that although all of the three arms of ER stress were activated, PERK deletion exhibited stronger protective effect against apoptosis compared with ATF6 and IRE1.^91^ On further investigation by subcellular fractionation, PERK was found as a component of mitochondria-associated endoplasmic reticulum membrane (MAM).^91, 92^ In other words, PERK is an MAM protein. MAM is described as the physical and functional contact site of ER and mitochondria, which is tightly packed with proteins with various functions including oxidative stress sensing, cell death regulation and so on.^93^ Thus, as an MAM protein, it is possible that PERK carries out more responsibilities in conducting death signals. However, more investigations should be implemented to address PERK-specific role as an MAM protein and its interactions with other MAM proteins.

The PERK/ATF4 signaling is now accepted as a typical pathway mediating ER stress-induced autophagy. It is believed that activated FoxO facilitates the expression of Bnip3, which displaces the autophagic effecter Beclin1 from Bcl-XL complexes to induce autophagy.^94^ A recent study pointed out that PERK could direct phosphorylation of FoxO transcription factor to promote FoxO activity.^95^ It is reasonable for us to speculate that PERK/FoxO could be an alternative PERK- associated pathway mediating ER stress-induced autophagy. Notably, PERK signaling is cell fate deciding in ER stress because of its autophagy–apoptosis switching role. As mentioned in above sections, p38 is tightly associated with the switching function of PERK. Are there other possible mechanisms? After calcium outflow is induced by PERK-mediated RyR2 opening, the calmodulin-dependent potein kinase kinase*β* (CAMPKK*β*) is activated,^96^ which subsequently induces AMPK signaling to affect mTOR-associated autophagy.^97^ Thus, CAMPKK*β* could also be treated as a potential key autophagy–apoptosis switching molecule in PERK signaling. However, these are only reasonable hypothesis, much more studies are still needed.

## Conclusion

According to the literature and our previous studies, although still not completely clear, PERK signaling pathway is potentially highly related with the types of human diseases including Alzheimer's disease,^98^ Parkinson's disease,^99^ Huntington's disease,^100^ encephalomyelitis,^101^ vascular calcification,^102^ arrhythmias,^64^ diabetic cardiomyopathy^91^ and so on. Clarification of basic mechanisms of ER stress-induced apoptosis, autophagy and the crosstalk between them is that these findings would be significant and benefit in interpreting pathological and pathophysiological mechanisms of certain diseases and finding novel therapeutic molecular targets.

## Figures and Tables

**Table 1:** Several molecules related with PERK-induced cell apoptosis

**Molecules**	**Upstream molecules**	**Effect of upstream molecules**	**Direct/indirect interact with PERK**	**Downstream molecules**	**Effect to downstream molecules**	**Consequence of activation**
eIF2*α*	PERK	Activation	Direct	ATF4	Activation	Proapoptosis
ATF4	PERK, eIF2*α*	Activation	Indirect	CHOP, Noxa	Activation	Proapoptosis
cIAPs	PERK, eIF2*α*, NAT1, DAP5	Activation	Unknown	Caspase-3, caspase-7, caspase-9	Inhibition	Antiapoptosis
TDAG51	PERK, eIF2*α*	Activation	Unknown	CD95/Fas	Activation	Proapoptosis
NRF	PERK	Activation	Direct	Bcl2	Activation	Antiapoptosis
TRIB3	PERK, ATF4, CHOP	Activation	Indirect	NF-*κ*B, Notch1	Activation	Proapoptosis
CaN	PERK	Activation	Direct	RyR2	Activation	Proapoptosis

**Table 2:** Several autophagic genes targeted by ER stress transcription factors

**ER stress transcription factor**	**Targeted autophagic gene**
ATF6	*DAPK1*^72^
XBP1	*Beclin1*,^73^ *Bcl2*^74^
ATF4	*Atg3*,^75^ *Atg12*,^75^ *Atg16*,^75^ *Map1*,^75^ *Beclin1*,^75^ *Gabarap12*^75^
CHOP	*Atg5*,^75^ *LC3*^75^
Coactivation of ATF4 and CHOP	*SQSTM1*,^75^ *Nbr1*,^75^ *Atg7*^75^
C/EBP*β*	*DAPK1*,^76^ *ULK1*,^77^ *Atg4B*^78^

## Abbreviations

ER: endoplasmic reticulum
ERAD: ER-associated degradation
ERQC: ER quality control
UPR: unfolded protein response
GRP: glucose-regulated protein
Hsp: heat shock protein
ATF: activating transcription factor
PERK: protein kinase R-like endoplasmic reticulum kinase
IRE: inositol requiring kinase
RIDD: regulated IRE1-dependent decay of mRNA
CHOP: C/BEP homologous protein
XBP: X-box binding protein
eIF: eukaryotic initiation factor
DR: death receptor
ERO: ER oxidase
ORF: open reading frames
CREB: cAMP-responsive element-binding protein
cIAP: cellular inhibitor of apoptosis
TDAG: T-cell death-associated gene
NRF: nuclear respiratory factor
TRIB: Tribbles homolog
CaN: calcineurin
FKBP: FK506-binding protein
RyR: ryanodine receptor
DAPK: death-associated protein kinase
mTOR: mammalian target of rapamycin
AMPK: AMP-activated protein kinase

## References

[bib1] 1Boelens J, Lust S, Offner F, Bracke ME, Vanhoecke BW. Review. The endoplasmic reticulum: a target for new anticancer drugs. In Vivo 2007; 21: 215–226. 17436569

[bib2] 2Gregor MF, Hotamisligil GS. Thematic review series: adipocyte biology. Adipocyte stress: the endoplasmic reticulum and metabolic disease. J Lipid Res 2007; 48: 1905–1914. 17699733 10.1194/jlr.R700007-JLR200

[bib3] 3Bollini R, Chrispeels MJ. The rough endoplasmic reticulum is the site of reserve-protein synthesis in developing Phaseolus vulgaris cotyledons. Planta 1979; 146: 487–501. 24318258 10.1007/BF00380865

[bib4] 4Rizzolo LJ, Kornfeld R. Post-translational protein modification in the endoplasmic reticulum. Demonstration of fatty acylase and deoxymannojirimycin-sensitive alpha-mannosidase activities. J Biol Chem 1988; 263: 9520–9525. 2967826

[bib5] 5Karnik AB, Thakore KN, Nigam SK, Babu KA, Lakkad BC, Bhatt DK et al. Studies on glucose-6-phosphatase, fructose-1,6-diphosphatase activity, glycogen distribution and endoplasmic reticulum changes during hexachlorocyclohexane induced hepatocarcinogenesis in pure inbred Swiss mice. Neoplasma 1981; 28: 575–584. 6171739

[bib6] 6Roitsch T, Lehle L. Post-translational translocation of polypeptides across the mammalian endoplasmic reticulum membrane is size and ribosome dependent. Eur J Biochem 1988; 174: 699–705. 3292239 10.1111/j.1432-1033.1988.tb14154.x

[bib7] 7Kondratyev M, Avezov E, Shenkman M, Groisman B, Lederkremer GZ. PERK-dependent compartmentalization of ERAD and unfolded protein response machineries during ER stress. Exp Cell Res 2007; 313: 3395–3407. 17707796 10.1016/j.yexcr.2007.07.006

[bib8] 8Hampton RY. ER stress response: getting the UPR hand on misfolded proteins. Curr Biol 2000; 10: R518–R521. 10898996 10.1016/s0960-9822(00)00583-2

[bib9] 9Kozutsumi Y, Segal M, Normington K, Gething MJ, Sambrook J. The presence of malfolded proteins in the endoplasmic reticulum signals the induction of glucose-regulated proteins. Nature 1988; 332: 462–464. 3352747 10.1038/332462a0

[bib10] 10Rao RV, Peel A, Logvinova A, del Rio G, Hermel E, Yokota T et al. Coupling endoplasmic reticulum stress to the cell death program: role of the ER chaperone GRP78. FEBS Lett 2002; 514: 122–128. 11943137 10.1016/s0014-5793(02)02289-5 PMC3971841

[bib11] 11Lajoie P, Moir RD, Willis IM, Snapp EL. Kar2p availability defines distinct forms of endoplasmic reticulum stress in living cells. Mol Biol Cell 2012; 23: 955–964. 22219379 10.1091/mbc.E11-12-0995 PMC3290652

[bib12] 12Vannuvel K, Renard P, Raes M, Arnould T. Functional and morphological impact of ER stress on mitochondria. J Cell Physiol 2013; 228: 1802–1818. 23629871 10.1002/jcp.24360

[bib13] 13Reddy RK, Mao C, Baumeister P, Austin RC, Kaufman RJ, Lee AS. Endoplasmic reticulum chaperone protein GRP78 protects cells from apoptosis induced by topoisomerase inhibitors: role of ATP binding site in suppression of caspase-7 activation. J Biol Chem 2003; 278: 20915–20924. 12665508 10.1074/jbc.M212328200

[bib14] 14Lee AS. The ER chaperone and signaling regulator GRP78/BiP as a monitor of endoplasmic reticulum stress. Methods 2005; 35: 373–381. 15804610 10.1016/j.ymeth.2004.10.010

[bib15] 15Rutkowski DT, Kaufman RJ. A trip to the ER: coping with stress. Trends Cell Biol 2004; 14: 20–28. 14729177 10.1016/j.tcb.2003.11.001

[bib16] 16Ye J, Rawson RB, Komuro R, Chen X, Dave UP, Prywes R et al. ER stress induces cleavage of membrane-bound ATF6 by the same proteases that process SREBPs. Mol Cell 2000; 6: 1355–1364. 11163209 10.1016/s1097-2765(00)00133-7

[bib17] 17Hirsch I, Weiwad M, Prell E, Ferrari DM. ERp29 deficiency affects sensitivity to apoptosis via impairment of the ATF6-CHOP pathway of stress response. Apoptosis 2014; 19: 801–815. 24370996 10.1007/s10495-013-0961-0

[bib18] 18Lee K, Tirasophon W, Shen X, Michalak M, Prywes R, Okada T et al. IRE1-mediated unconventional mRNA splicing and S2P-mediated ATF6 cleavage merge to regulate XBP1 in signaling the unfolded protein response. Genes Dev 2002; 16: 452–466. 11850408 10.1101/gad.964702 PMC155339

[bib19] 19Calfon M, Zeng H, Urano F, Till JH, Hubbard SR, Harding HP et al. IRE1 couples endoplasmic reticulum load to secretory capacity by processing the XBP-1 mRNA. Nature 2002; 415: 92–96. 11780124 10.1038/415092a

[bib20] 20Malhi H, Kaufman RJ. Endoplasmic reticulum stress in liver disease. J Hepatol 2011; 54: 795–809. 21145844 10.1016/j.jhep.2010.11.005 PMC3375108

[bib21] 21Sano R, Reed JC. ER stress-induced cell death mechanisms. Biochim Biophys Acta 2013; 1833: 3460–3470. 23850759 10.1016/j.bbamcr.2013.06.028 PMC3834229

[bib22] 22Hollien J, Lin JH, Li H, Stevens N, Walter P, Weissman JS. Regulated Ire1-dependent decay of messenger RNAs in mammalian cells. J Cell Biol 2009; 186: 323–331. 19651891 10.1083/jcb.200903014 PMC2728407

[bib23] 23Donnelly N, Gorman AM, Gupta S, Samali A. The eIF2alpha kinases: their structures and functions. Cell Mol Life Sci 2013; 70: 3493–3511. 23354059 10.1007/s00018-012-1252-6 PMC11113696

[bib24] 24Marciniak SJ, Garcia-Bonilla L, Hu J, Harding HP, Ron D. Activation-dependent substrate recruitment by the eukaryotic translation initiation factor 2 kinase PERK. J Cell Biol 2006; 172: 201–209. 16418533 10.1083/jcb.200508099 PMC2063550

[bib25] 25Zhao Y, Guo Z, Lin X, Zhou L, Okoro EU, Fan G et al. Apolipoprotein E-deficient lipoproteins induce foam cell formation by activation of PERK-EIF-2alpha signaling cascade. J Bioanal Biomed 2010; 2: 113–120. 21552349 10.4172/1948-593x.1000033 PMC3086298

[bib26] 26Locker N, Easton LE, Lukavsky PJ. HCV and CSFV IRES domain II mediate eIF2 release during 80 S ribosome assembly. EMBO J 2007; 26: 795–805. 17255934 10.1038/sj.emboj.7601549 PMC1794401

[bib27] 27Vattem KM, Wek RC. Reinitiation involving upstream ORFs regulates ATF4 mRNA translation in mammalian cells. Proc Natl Acad Sci USA 2004; 101: 11269–11274. 15277680 10.1073/pnas.0400541101 PMC509193

[bib28] 28Wek RC, Cavener DR. Translational control and the unfolded protein response. Antioxid Redox Signal 2007; 9: 2357–2371. 17760508 10.1089/ars.2007.1764

[bib29] 29Saito A, Ochiai K, Kondo S, Tsumagari K, Murakami T, Cavener DR et al. Endoplasmic reticulum stress response mediated by the PERK-eIF2(alpha)-ATF4 pathway is involved in osteoblast differentiation induced by BMP2. J Biol Chem 2011; 286: 4809–4818. 21135100 10.1074/jbc.M110.152900 PMC3039352

[bib30] 30Cui W, Li J, Ron D, Sha B. The structure of the PERK kinase domain suggests the mechanism for its activation. Acta Crystallogr D 2011; 67: 423–428. 21543844 10.1107/S0907444911006445 PMC3087621

[bib31] 31Fang F, Gong PS, Song XF, Gong SL, Wang ZC. Low-dose radiation induces endoplasmic reticulum stress and activates PERK-CHOP signaling pathway in mouse testicular cells]. Zhonghua Nan Ke Xue 2012; 18: 777–782. 23193662

[bib32] 32Yamaguchi H, Wang HG. CHOP is involved in endoplasmic reticulum stress-induced apoptosis by enhancing DR5 expression in human carcinoma cells. J Biol Chem 2004; 279: 45495–45502. 15322075 10.1074/jbc.M406933200

[bib33] 33Frand AR, Kaiser CA. The ERO1 gene of yeast is required for oxidation of protein dithiols in the endoplasmic reticulum. Mol Cell 1998; 1: 161–170. 9659913 10.1016/s1097-2765(00)80017-9

[bib34] 34Szegezdi E, Fitzgerald U, Samali A. Caspase-12 and ER-stress-mediated apoptosis: the story so far. Ann NY Acad Sci 2003; 1010: 186–194. 15033718 10.1196/annals.1299.032

[bib35] 35Binet F, Chiasson S, Girard D. Evidence that endoplasmic reticulum (ER) stress and caspase-4 activation occur in human neutrophils. Biochem Biophys Res Commun 2010; 391: 18–23. 19878647 10.1016/j.bbrc.2009.10.141

[bib36] 36Martinez JA, Zhang Z, Svetlov SI, Hayes RL, Wang KK, Larner SF. Calpain and caspase processing of caspase-12 contribute to the ER stress-induced cell death pathway in differentiated PC12 cells. Apoptosis 2010; 15: 1480–1493. 20640600 10.1007/s10495-010-0526-4

[bib37] 37Thompson SR, Gulyas KD, Sarnow P. Internal initiation in *Saccharomyces cerevisiae* mediated by an initiator tRNA/eIF2-independent internal ribosome entry site element. Proc Natl Acad Sci USA 2001; 98: 12972–12977. 11687653 10.1073/pnas.241286698 PMC60809

[bib38] 38Chambers JE, Dalton LE, Clarke HJ, Malzer E, Dominicus CS, Patel V et al. Actin dynamics tune the integrated stress response by regulating eukaryotic initiation factor 2alpha dephosphorylation. eLife 2015; 4: e04872. 10.7554/eLife.04872 PMC4394351 25774599

[bib39] 39Chen Y, Gao H, Yin Q, Chen L, Dong P, Zhang X et al. ER stress activating ATF4/CHOP-TNF-alpha signaling pathway contributes to alcohol-induced disruption of osteogenic lineage of multipotential mesenchymal stem cell. Cell Physiol Biochem 2013; 32: 743–754. 24080827 10.1159/000354476

[bib40] 40Su N, Kilberg MS. C/EBP homology protein (CHOP) interacts with activating transcription factor 4 (ATF4) and negatively regulates the stress-dependent induction of the asparagine synthetase gene. J Biol Chem 2008; 283: 35106–35117. 18940792 10.1074/jbc.M806874200 PMC2596379

[bib41] 41Cao J, Dai DL, Yao L, Yu HH, Ning B, Zhang Q et al. Saturated fatty acid induction of endoplasmic reticulum stress and apoptosis in human liver cells via the PERK/ATF4/CHOP signaling pathway. Mol Cell Biochem 2012; 364: 115–129. 22246806 10.1007/s11010-011-1211-9

[bib42] 42Qing G, Li B, Vu A, Skuli N, Walton ZE, Liu X et al. ATF4 regulates MYC-mediated neuroblastoma cell death upon glutamine deprivation. Cancer Cell 2012; 22: 631–644. 23153536 10.1016/j.ccr.2012.09.021 PMC3510660

[bib43] 43Foster KA, Jane EP, Premkumar DR, Morales A, Pollack IF. Co-administration of ABT-737 and SAHA induces apoptosis, mediated by Noxa upregulation, Bax activation and mitochondrial dysfunction in PTEN-intact malignant human glioma cell lines. J Neurooncol 2014; 120: 459–472. 25139025 10.1007/s11060-014-1575-2

[bib44] 44Dong Z, Wang JZ, Yu F, Venkatachalam MA. Apoptosis-resistance of hypoxic cells: multiple factors involved and a role for IAP-2. Am J Pathol 2003; 163: 663–671. 12875985 10.1016/S0002-9440(10)63693-0 PMC1868200

[bib45] 45de Graaf AO, van Krieken JH, Tonnissen E, Wissink W, van de Locht L, Overes I et al. Expression of C-IAP1, C-IAP2 and SURVIVIN discriminates different types of lymphoid malignancies. Br J Haematol 2005; 130: 852–859. 16156855 10.1111/j.1365-2141.2005.05690.x

[bib46] 46Hamanaka RB, Bobrovnikova-Marjon E, Ji X, Liebhaber SA, Diehl JA. PERK-dependent regulation of IAP translation during ER stress. Oncogene 2009; 28: 910–920. 19029953 10.1038/onc.2008.428 PMC2642534

[bib47] 47Warnakulasuriyarachchi D, Cerquozzi S, Cheung HH, Holcik M. Translational induction of the inhibitor of apoptosis protein HIAP2 during endoplasmic reticulum stress attenuates cell death and is mediated via an inducible internal ribosome entry site element. J Biol Chem 2004; 279: 17148–17157. 14960583 10.1074/jbc.M308737200

[bib48] 48Fulda S, Vucic D, Targeting IAP. proteins for therapeutic intervention in cancer. Nat Rev Drug Discov 2012; 11: 109–124. 22293567 10.1038/nrd3627

[bib49] 49Park ES, Kim J, Ha TU, Choi JS, Soo Hong K, Rho J. TDAG51 deficiency promotes oxidative stress-induced apoptosis through the generation of reactive oxygen species in mouse embryonic fibroblasts. Exp Mol Med 2013; 45: e35. 23928855 10.1038/emm.2013.67 PMC3789259

[bib50] 50Hossain GS, van Thienen JV, Werstuck GH, Zhou J, Sood SK, Dickhout JG et al. TDAG51 is induced by homocysteine, promotes detachment-mediated programmed cell death, and contributes to the cevelopment of atherosclerosis in hyperhomocysteinemia. J Biol Chem 2003; 278: 30317–30327. 12738777 10.1074/jbc.M212897200

[bib51] 51Zhou J, Lhotak S, Hilditch BA, Austin RC. Activation of the unfolded protein response occurs at all stages of atherosclerotic lesion development in apolipoprotein E-deficient mice. Circulation 2005; 111: 1814–1821. 15809369 10.1161/01.CIR.0000160864.31351.C1

[bib52] 52Baird L, Dinkova-Kostova AT. The cytoprotective role of the Keap1-Nrf2 pathway. Arch Toxicol 2011; 85: 241–272. 21365312 10.1007/s00204-011-0674-5

[bib53] 53Cullinan SB, Zhang D, Hannink M, Arvisais E, Kaufman RJ, Diehl JA. Nrf2 is a direct PERK substrate and effector of PERK-dependent cell survival. Mol Cell Biol 2003; 23: 7198–7209. 14517290 10.1128/MCB.23.20.7198-7209.2003 PMC230321

[bib54] 54Del Vecchio CA, Feng Y, Sokol ES, Tillman EJ, Sanduja S, Reinhardt F et al. De-differentiation confers multidrug resistance via noncanonical PERK-Nrf2 signaling. PLoS Biol 2014; 12: e1001945. 25203443 10.1371/journal.pbio.1001945 PMC4159113

[bib55] 55Digaleh H, Kiaei M, Khodagholi F. Nrf2 and Nrf1 signaling and ER stress crosstalk: implication for proteasomal degradation and autophagy. Cell Mol Life Sci 2013; 70: 4681–4694. 23800989 10.1007/s00018-013-1409-y PMC11113484

[bib56] 56Zhang B, Wang XQ, Chen HY, Liu BH. Involvement of the Nrf2 pathway in the regulation of pterostilbene-induced apoptosis in HeLa cells via ER stress. J Pharmacol Sci 2014; 126: 216–229. 25341683 10.1254/jphs.14028fp

[bib57] 57Cullinan SB, Diehl JA. Coordination of ER and oxidative stress signaling: the PERK/Nrf2 signaling pathway. Int J Biochem Cell Biol 2006; 38: 317–332. 16290097 10.1016/j.biocel.2005.09.018

[bib58] 58Qian B, Wang H, Men X, Zhang W, Cai H, Xu S et al. TRIB3 [corrected] is implicated in glucotoxicity- and endoplasmic reticulum-stress-induced [corrected] beta-cell apoptosis. J Endocrinol 2008; 199: 407–416. 18818302 10.1677/JOE-08-0331

[bib59] 59Ishikawa F, Akimoto T, Yamamoto H, Araki Y, Yoshie T, Mori K et al. Gene expression profiling identifies a role for CHOP during inhibition of the mitochondrial respiratory chain. J Biochem 2009; 146: 123–132. 19304788 10.1093/jb/mvp052

[bib60] 60Kim EJ, Lee YJ, Kang S, Lim YB. Ionizing radiation activates PERK/eIF2alpha/ATF4 signaling via ER stress-independent pathway in human vascular endothelial cells. Int J Radiat Biol 2014; 90: 306–312. 24456547 10.3109/09553002.2014.886793

[bib61] 61Leaver HA, Schou AC, Rizzo MT, Prowse CV. Calcium-sensitive mitochondrial membrane potential in human platelets and intrinsic signals of cell death. Platelets 2006; 17: 368–377. 16973497 10.1080/09537100600757216

[bib62] 62Du Y, Zhao J, Li X, Jin S, Ma WL, Mu Q et al. Dissociation of FK506-binding protein 12.6 kD from ryanodine receptor in bronchial smooth muscle cells in airway hyperresponsiveness in asthma. Am J Respir Cell Mol Biol 2014; 50: 398–408. 24053175 10.1165/rcmb.2013-0222OC PMC5800890

[bib63] 63Hammadi M, Oulidi A, Gackiere F, Katsogiannou M, Slomianny C, Roudbaraki M et al. Modulation of ER stress and apoptosis by endoplasmic reticulum calcium leak via translocon during unfolded protein response: involvement of GRP78. FASEB J 2013; 27: 1600–1609. 23322163 10.1096/fj.12-218875

[bib64] 64Liu Z, Cai H, Zhu H, Toque H, Zhao N, Qiu C et al. Protein kinase RNA-like endoplasmic reticulum kinase (PERK)/calcineurin signaling is a novel pathway regulating intracellular calcium accumulation which might be involved in ventricular arrhythmias in diabetic cardiomyopathy. Cell Signal 2014; 26: 2591–2600. 25152364 10.1016/j.cellsig.2014.08.015

[bib65] 65Ouyang L, Shi Z, Zhao S, Wang FT, Zhou TT, Liu B et al. Programmed cell death pathways in cancer: a review of apoptosis, autophagy and programmed necrosis. Cell Prolif 2012; 45: 487–498. 23030059 10.1111/j.1365-2184.2012.00845.x PMC6496669

[bib66] 66Mijaljica D, Prescott M, Devenish RJ. Endoplasmic reticulum and Golgi complex: contributions to, and turnover by, autophagy. Traffic 2006; 7: 1590–1595. 17040485 10.1111/j.1600-0854.2006.00495.x

[bib67] 67Yorimitsu T, Nair U, Yang Z, Klionsky DJ. Endoplasmic reticulum stress triggers autophagy. J Biol Chem 2006; 281: 30299–30304. 16901900 10.1074/jbc.M607007200 PMC1828866

[bib68] 68Yorimitsu T, Klionsky DJ. Endoplasmic reticulum stress: a new pathway to induce autophagy. Autophagy 2007; 3: 160–162. 17204854 10.4161/auto.3653

[bib69] 69Salazar M, Carracedo A, Salanueva IJ, Hernandez-Tiedra S, Lorente M, Egia A et al. Cannabinoid action induces autophagy-mediated cell death through stimulation of ER stress in human glioma cells. J Clin Invest 2009; 119: 1359–1372. 19425170 10.1172/JCI37948 PMC2673842

[bib70] 70Gozuacik D, Bialik S, Raveh T, Mitou G, Shohat G, Sabanay H et al. DAP-kinase is a mediator of endoplasmic reticulum stress-induced caspase activation and autophagic cell death. Cell Death Differ 2008; 15: 1875–1886. 18806755 10.1038/cdd.2008.121

[bib71] 71Rubiolo JA, Lopez-Alonso H, Martinez P, Millan A, Cagide E, Vieytes MR et al. Yessotoxin induces ER-stress followed by autophagic cell death in glioma cells mediated by mTOR and BNIP3. Cell Signal 2014; 26: 419–432. 24511615

[bib72] 72Gade P, Manjegowda SB, Nallar SC, Maachani UB, Cross AS, Kalvakolanu DV. Regulation of the death-associated protein kinase 1 expression and autophagy via ATF6 requires apoptosis signal-regulating kinase 1. Mol Cell Biol 2014; 34: 4033–4048. 25135476 10.1128/MCB.00397-14 PMC4386459

[bib73] 73Margariti A, Li H, Chen T, Martin D, Vizcay-Barrena G, Alam S et al. XBP1 mRNA splicing triggers an autophagic response in endothelial cells through BECLIN-1 transcriptional activation. J Biol Chem 2013; 288: 859–872. 23184933 10.1074/jbc.M112.412783 PMC3543035

[bib74] 74Gomez BP, Riggins RB, Shajahan AN, Klimach U, Wang A, Crawford AC et al. Human X-box binding protein-1 confers both estrogen independence and antiestrogen resistance in breast cancer cell lines. FASEB J 2007; 21: 4013–4027. 17660348 10.1096/fj.06-7990com

[bib75] 75B'Chir W, Maurin AC, Carraro V, Averous J, Jousse C, Muranishi Y et al. The eIF2alpha/ATF4 pathway is essential for stress-induced autophagy gene expression. Nucleic Acids Res 2013; 41: 7683–7699. 23804767 10.1093/nar/gkt563 PMC3763548

[bib76] 76Gade P, Roy SK, Li H, Nallar SC, Kalvakolanu DV. Critical role for transcription factor C/EBP-beta in regulating the expression of death-associated protein kinase 1. Mol Cell Biol 2008; 28: 2528–2548. 18250155 10.1128/MCB.00784-07 PMC2293111

[bib77] 77Ma D, Panda S, Lin JD. Temporal orchestration of circadian autophagy rhythm by C/EBPbeta. EMBO J 2011; 30: 4642–4651. 21897364 10.1038/emboj.2011.322 PMC3243590

[bib78] 78Guo L, Huang JX, Liu Y, Li X, Zhou SR, Qian SW et al. Transactivation of Atg4b by C/EBPbeta promotes autophagy to facilitate adipogenesis. Mol Cell Biol 2013; 33: 3180–3190. 23754749 10.1128/MCB.00193-13 PMC3753907

[bib79] 79Lee H, Noh JY, Oh Y, Kim Y, Chang JW, Chung CW et al. IRE1 plays an essential role in ER stress-mediated aggregation of mutant huntingtin via the inhibition of autophagy flux. Hum Mol Genet 2012; 21: 101–114. 21954231 10.1093/hmg/ddr445

[bib80] 80Choi MS, Kim Y, Jung JY, Yang SH, Lee TR, Shin DW. Resveratrol induces autophagy through death-associated protein kinase 1 (DAPK1) in human dermal fibroblasts under normal culture conditions. Exp Dermatol 2013; 22: 491–494. 23800064 10.1111/exd.12175

[bib81] 81Gade P, Ramachandran G, Maachani UB, Rizzo MA, Okada T, Prywes R et al. An IFN-gamma-stimulated ATF6-C/EBP-beta-signaling pathway critical for the expression of death associated protein kinase 1 and induction of autophagy. Proc Natl Acad Sci USA 2012; 109: 10316–10321. 22699507 10.1073/pnas.1119273109 PMC3387052

[bib82] 82Kouroku Y, Fujita E, Tanida I, Ueno T, Isoai A, Kumagai H et al. ER stress (PERK/eIF2alpha phosphorylation) mediates the polyglutamine-induced LC3 conversion, an essential step for autophagy formation. Cell Death Differ 2007; 14: 230–239. 16794605 10.1038/sj.cdd.4401984

[bib83] 83Rzymski T, Milani M, Pike L, Buffa F, Mellor HR, Winchester L et al. Regulation of autophagy by ATF4 in response to severe hypoxia. Oncogene 2010; 29: 4424–4435. 20514020 10.1038/onc.2010.191

[bib84] 84Avivar-Valderas A, Bobrovnikova-Marjon E, Alan Diehl J, Bardeesy N, Debnath J, Aguirre-Ghiso JA. Regulation of autophagy during ECM detachment is linked to a selective inhibition of mTORC1 by PERK. Oncogene 2013; 32: 4932–4940. 23160380 10.1038/onc.2012.512 PMC3600386

[bib85] 85Zarogoulidis P, Lampaki S, Turner JF, Huang H, Kakolyris S, Syrigos K et al. mTOR pathway: a current, up-to-date mini-review (Review). Oncol Lett 2014; 8: 2367–2370. 25360163 10.3892/ol.2014.2608 PMC4214394

[bib86] 86Dunlop EA, Tee AR. mTOR and autophagy: a dynamic relationship governed by nutrients and energy. Semin Cell Dev Biol 2014; 36: 121–129. 25158238 10.1016/j.semcdb.2014.08.006

[bib87] 87Shi WY, Xiao D, Wang L, Dong LH, Yan ZX, Shen ZX et al. Therapeutic metformin/AMPK activation blocked lymphoma cell growth via inhibition of mTOR pathway and induction of autophagy. Cell Death Dis 2012; 3: e275. 22378068 10.1038/cddis.2012.13 PMC3317343

[bib88] 88Jiang Q, Li F, Shi K, Wu P, An J, Yang Y et al. Involvement of p38 in signal switching from autophagy to apoptosis via the PERK/eIF2alpha/ATF4 axis in selenite-treated NB4 cells. Cell Death Dis 2014; 5: e1270. 24874742 10.1038/cddis.2014.200 PMC4047911

[bib89] 89Jiang Q, Li F, Shi K, Wu P, An J, Yang Y et al. ATF4 activation by the p38MAPK-eIF4E axis mediates apoptosis and autophagy induced by selenite in Jurkat cells. FEBS Lett 2013; 587: 2420–2429. 23792164 10.1016/j.febslet.2013.06.011

[bib90] 90Adachi S, Yasuda I, Nakashima M, Yamauchi T, Yamauchi J, Natsume H et al. HSP90 inhibitors induce desensitization of EGF receptor via p38 MAPK-mediated phosphorylation at Ser1046/1047 in human pancreatic cancer cells. Oncol Rep 2010; 23: 1709–1714. 20428829 10.3892/or_00000815

[bib91] 91Liu ZW, Zhu HT, Chen KL, Dong X, Wei J, Qiu C et al. Protein kinase RNA-like endoplasmic reticulum kinase (PERK) signaling pathway plays a major role in reactive oxygen species (ROS)-mediated endoplasmic reticulum stress-induced apoptosis in diabetic cardiomyopathy. Cardiovasc Diabetol 2013; 12: 158. 24180212 10.1186/1475-2840-12-158 PMC4176998

[bib92] 92Verfaillie T, Rubio N, Garg AD, Bultynck G, Rizzuto R, Decuypere JP et al. PERK is required at the ER–mitochondrial contact sites to convey apoptosis after ROS-based ER stress. Cell Death Differ 2012; 19: 1880–1891. 22705852 10.1038/cdd.2012.74 PMC3469056

[bib93] 93Pinton P, Giorgi C, Missiroli S, Patergnani S, Duszynski J, Wieckowski M. Mitochondria-associated membranes (MAMs): composition, molecular mechanisms and physiopathological implications. Antioxid Redox Signal 2015; 22: 995–1019. 25557408 10.1089/ars.2014.6223

[bib94] 94Lin A, Yao J, Zhuang L, Wang D, Han J, Lam EW et al. The FoxO-BNIP3 axis exerts a unique regulation of mTORC1 and cell survival under energy stress. Oncogene 2014; 33: 3183–3194. 23851496 10.1038/onc.2013.273 PMC4365448

[bib95] 95Zhang W, Hietakangas V, Wee S, Lim SC, Gunaratne J, Cohen SM. ER stress potentiates insulin resistance through PERK-mediated FOXO phosphorylation. Genes Dev 2013; 27: 441–449. 23431056 10.1101/gad.201731.112 PMC3589560

[bib96] 96Wen L, Chen Z, Zhang F, Cui X, Sun W, Geary GG et al. Ca^2+^/calmodulin-dependent protein kinase kinase beta phosphorylation of Sirtuin 1 in endothelium is atheroprotective. Proc Natl Acad Sci USA 2013; 110: E2420-7. 23754392 10.1073/pnas.1309354110 PMC3696824

[bib97] 97Yang S, Wang J. Estrogen activates AMP-activated protein kinase in human endothelial cells via ERbeta/Ca/calmodulin-dependent protein kinase kinase beta pathway. Cell Biochem Biophys 2015; 24: 24. 10.1007/s12013-015-0521-z 25616441

[bib98] 98Ma T, Trinh MA, Wexler AJ, Bourbon C, Gatti E, Pierre P et al. Suppression of eIF2alpha kinases alleviates Alzheimer's disease-related plasticity and memory deficits. Nat Neurosci 2013; 16: 1299–1305. 23933749 10.1038/nn.3486 PMC3756900

[bib99] 99Hashida K, Kitao Y, Sudo H, Awa Y, Maeda S, Mori K et al. ATF6alpha promotes astroglial activation and neuronal survival in a chronic mouse model of Parkinson's disease. PLoS One 2012; 7: e47950. 23112876 10.1371/journal.pone.0047950 PMC3480445

[bib100] 100Vidal RL, Hetz C. Crosstalk between the UPR and autophagy pathway contributes to handling cellular stress in neurodegenerative disease. Autophagy 2012; 8: 970–972. 22617512 10.4161/auto.20139 PMC3427262

[bib101] 101Meares GP, Liu Y, Rajbhandari R, Qin H, Nozell SE, Mobley JA et al. PERK-dependent activation of JAK1 and STAT3 contributes to endoplasmic reticulum stress-induced inflammation. Mol Cell Biol 2014; 34: 3911–3925. 25113558 10.1128/MCB.00980-14 PMC4187715

[bib102] 102Masuda M, Miyazaki-Anzai S, Levi M, Ting TC, Miyazaki M. PERK-eIF2alpha-ATF4-CHOP signaling contributes to TNFalpha-induced vascular calcification. J Am Heart Assoc 2013; 2: e000238. 24008080 10.1161/JAHA.113.000238 PMC3835225

